# Supplementary material for: The effects of seasonal climate variability on dengue annual incidence in Hong Kong: A modelling study
Source: Sci Rep. 2020 Mar 9;10:4297. doi: 10.1038/s41598-020-60309-7 (PMC7062697; doi:10.1038/s41598-020-60309-7)
Supplement: Supplementary file 1 — Supplementary Information1. [file 41598_2020_60309_MOESM1_ESM.pdf]

The effects of seasonal climate variability on dengue annual incidence in Hong Kong: A modelling study

Hsiang-Yu Yuan[1,*], Jingbo Liang[1], Pei-Sheng Lin[2,3], Kathleen Sucipto[1], Mesfin Mengesha Tsegaye[1], Tzai-Hung Wen[4], Susanne Pfeiffer[5], and Dirk Pfeiffer[6]

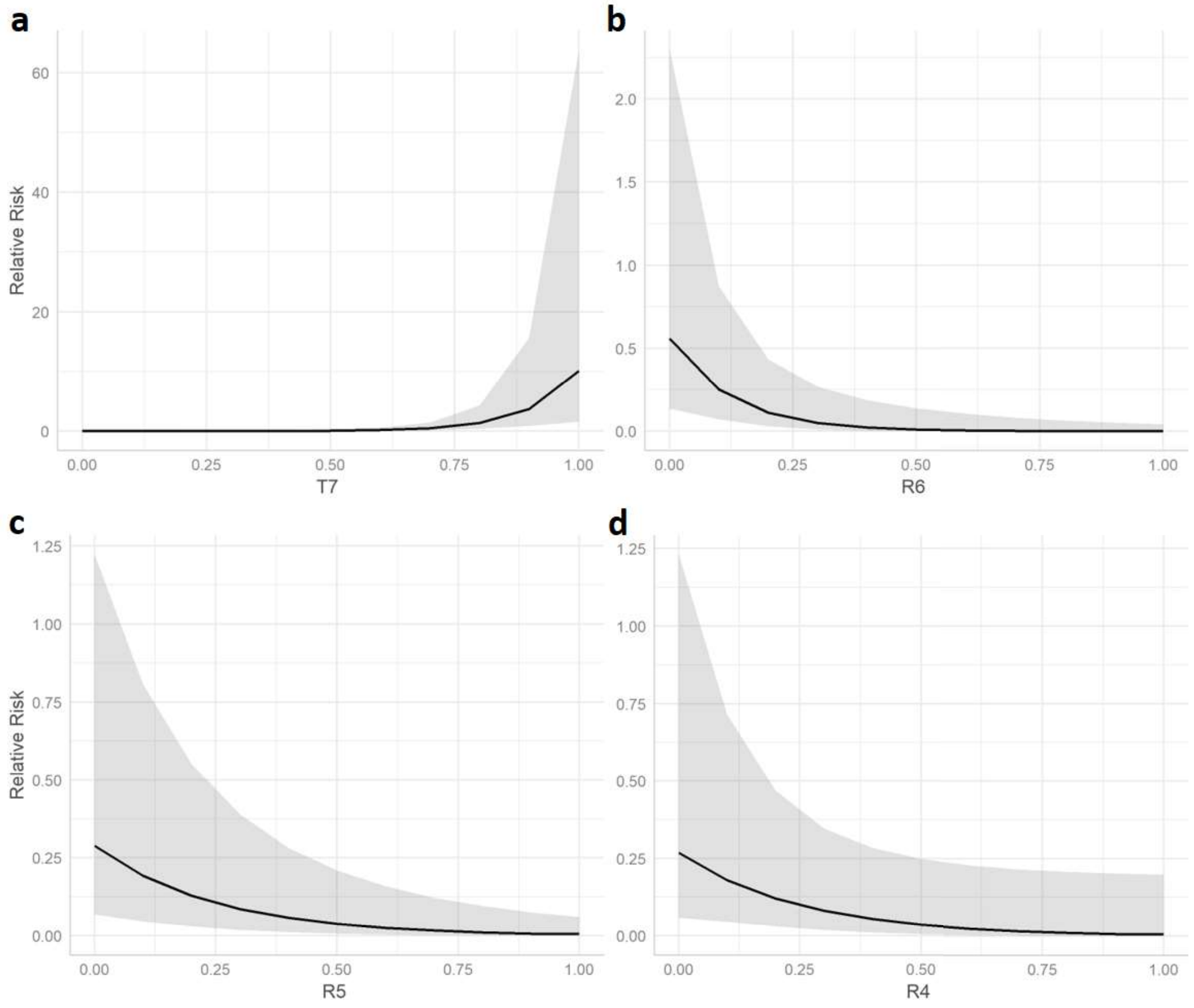

Figure S1. The marginal effects of the 4 predictors with the greatest magnitude of correlation coefficients on the relative risk (RR). The RR is defined as the predicted number of annual cases divided by the average number of annual cases over all years from 2002 to 2018. The marginal effects of (a) T_7, (b) R_6, (c) R_5 and (d) R_4 are included.

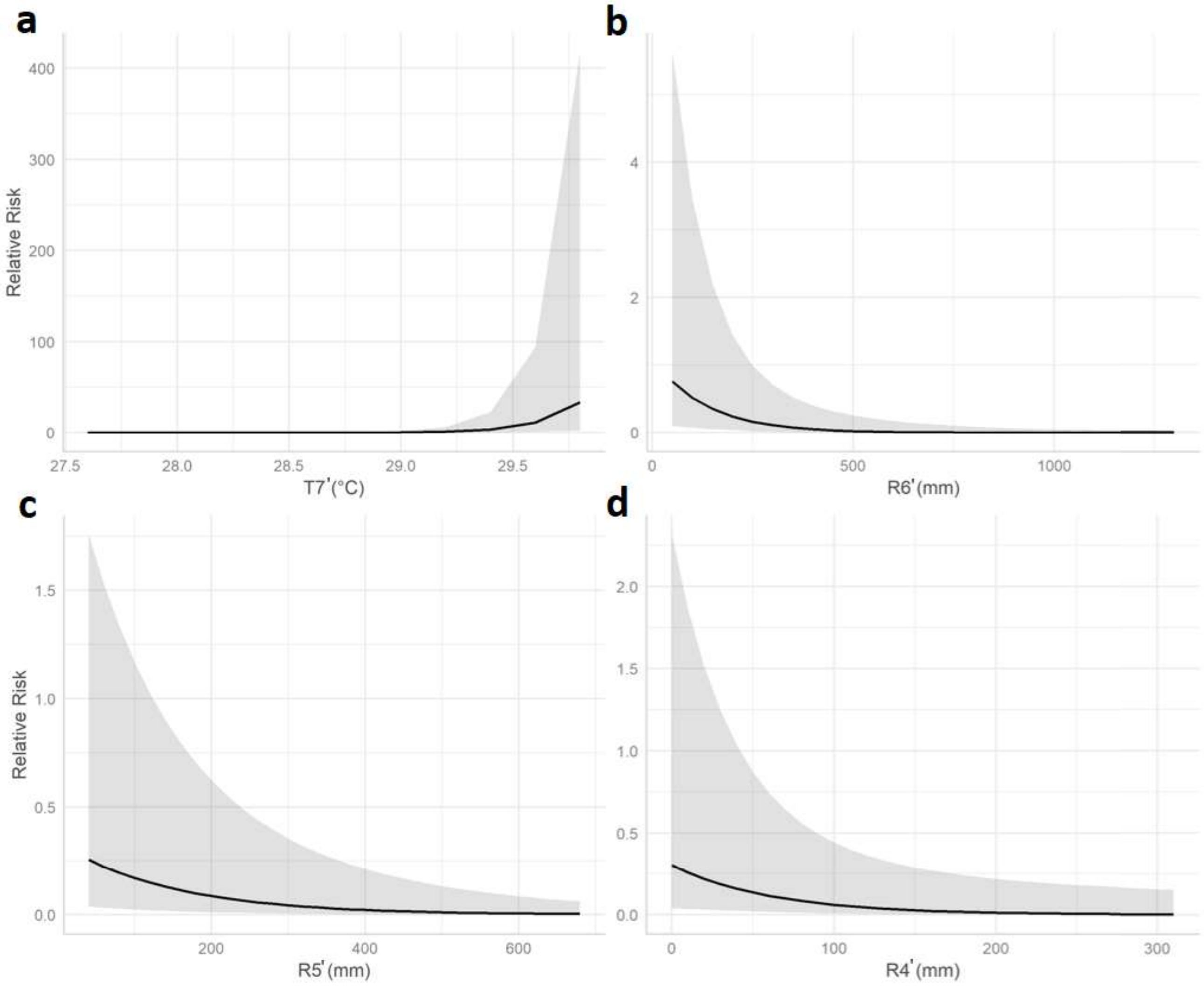

Figure S2. The marginal effects of the 4 predictors, using the original weather data, with the greatest magnitude of correlation coefficients on the relative risk (RR). The RR is defined as the number of annual cases divided by the average number of annual cases over all years from 2002 to 2018. The marginal effects of (a) T_7', (b) R_6', (c) R_5' and (d) R_4' are included. Note that the range of each predictor is in the original units but not the standardised ratio for easier comparison with the actual monthly climate data.

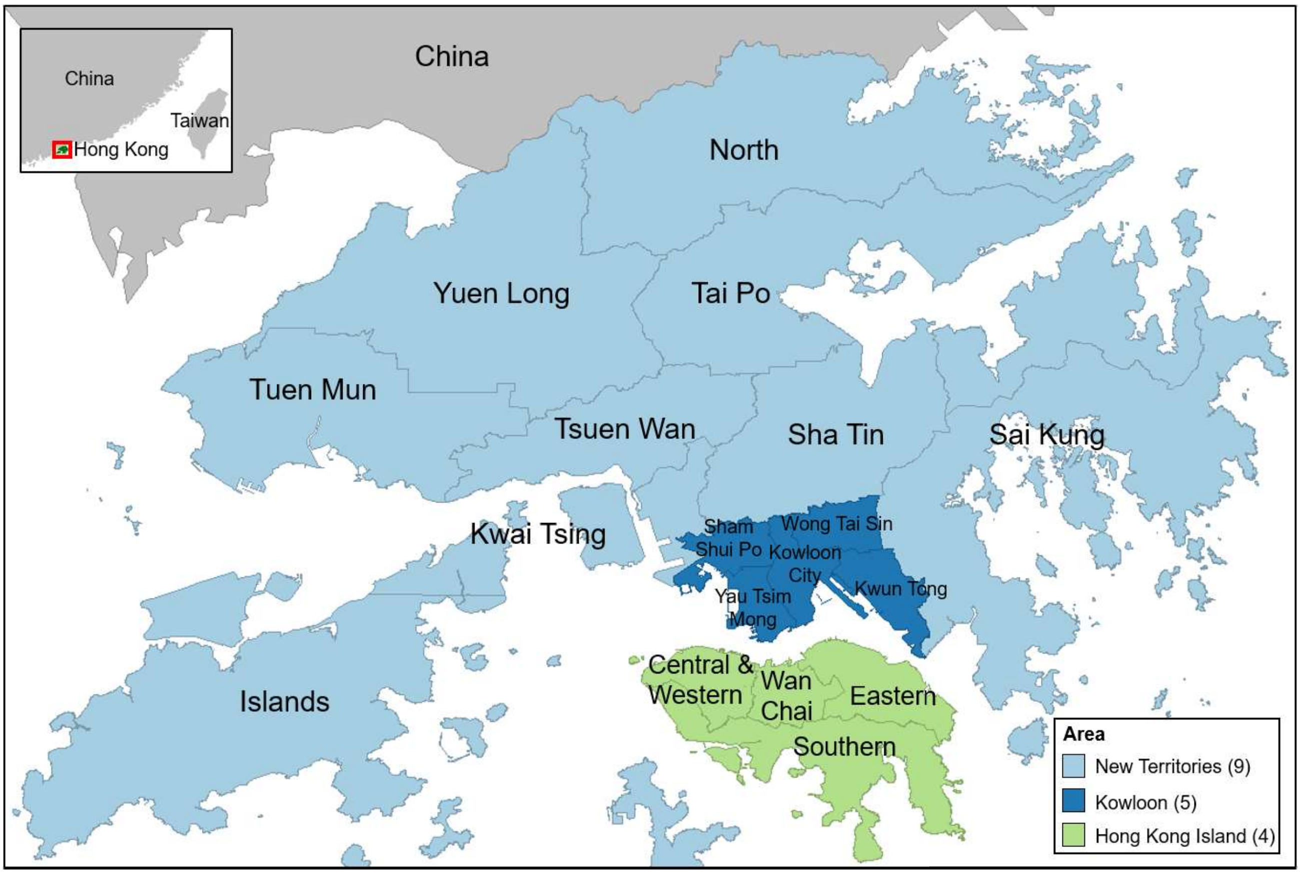

Figure S3. The official districts and regions of Hong Kong. Numbers in the legends indicate the number of districts belonging to each region.

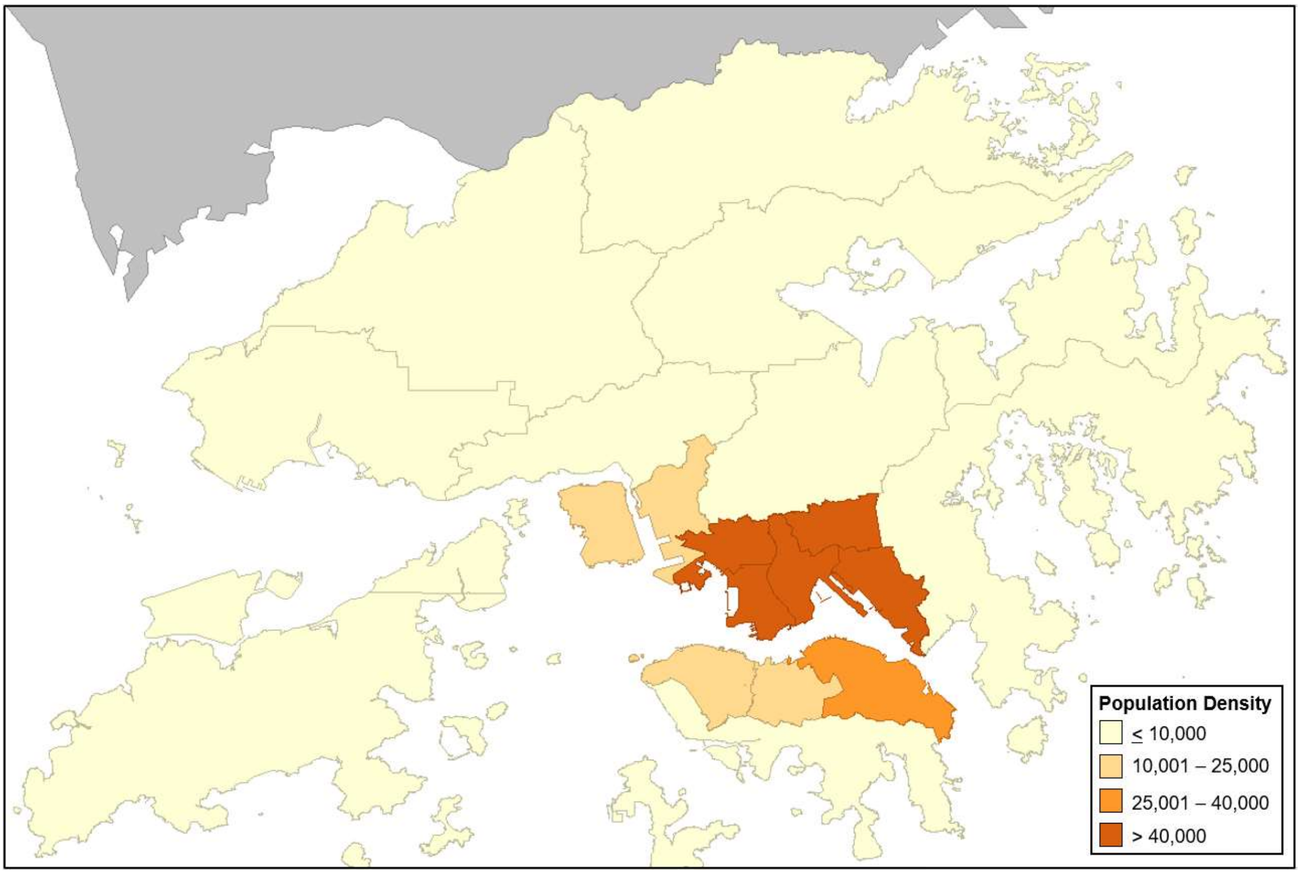

Figure S4. Hong Kong population density map. Density is defined as the total number of population divided by area in km^2.

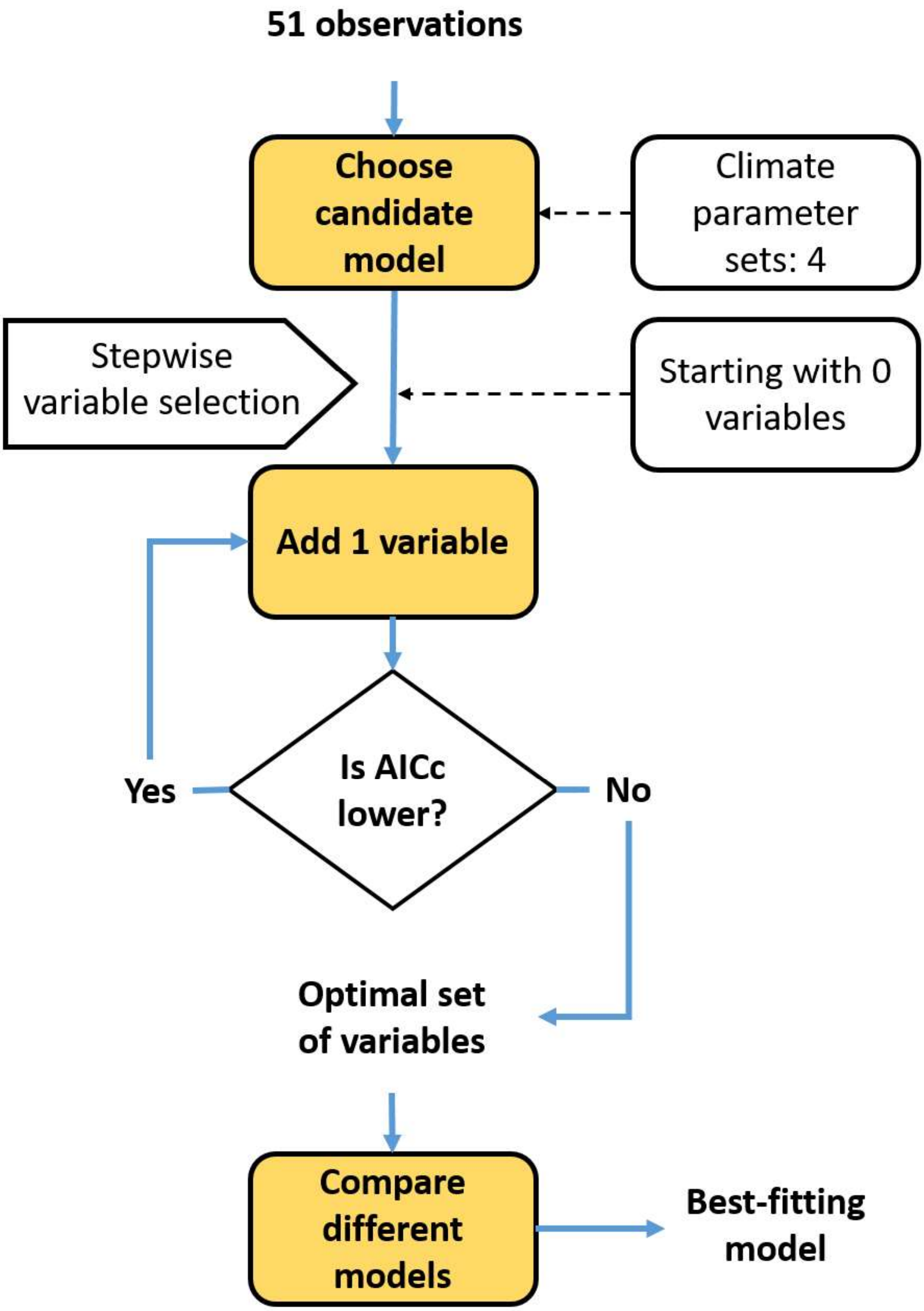

Figure S5. Flowchart for selecting the best-fitting model using a two-step procedure. The procedure involves variable selection for a candidate model with a climate parameter set as the first step and model comparison between four different climate parameter sets as the second step. The initial data includes a total of 51 observations in the pre-defined areas. The stepwise algorithm based on the Akaike Information Criterion with a correction (AICc) is used to produce the optimal set of variables.

Table S1. The variable selection stages of the stepwise forward selection algorithm with AICc for each set of monthly climate predictors. T_m and R_m denote the temperature and rainfall predictors in the mth month of the year. The order of the rows indicates the AICc results at each selection step. The order of the columns (from the left to right) represents the sequence of the variables that have been selected. A newly selected variable is listed to the right of the last variable. The optimal set of variables is listed for each climate parameter combination set (as in subtable i to iv).

(i) Monthly mean temperature (T_{mean}) and monthly total rainfall (R_{tot})

Variables								$AICc$
0	T_5	T_3	T_7	T_4	R_5	R_6	R_4	
0.138								266.79
-2.531	4.858							169.03
-4.732	3.454	4.372						139.49
-7.019	2.982	5.827	2.807					130.95
-10.903	2.475	6.070	6.043	3.323				116.78
-10.626	0.670	6.284	7.366	4.325	-2.311			113.31
-9.172	1.543	5.852	8.273	3.222	-3.292	-5.158		110.30
-6.200	1.210	3.594	10.072	2.542	-4.029	-8.047	-4.002	107.84

(ii) Monthly mean temperature (T_{mean}) and monthly maximum rainfall (R_{max})

Variables								$AICc$
0	T_5	T_3	T_7	T_4	R_5	R_4	R_8	
0.138								266.79
-2.531	4.858							169.03
-4.732	3.454	4.372						139.49
-7.019	2.982	5.827	2.807					130.95
-10.903	2.475	6.070	6.043	3.323				116.78
-9.751	1.644	5.315	6.438	3.578	-2.051			114.70
-6.544	0.873	3.406	6.860	2.720	-2.719	-5.613		110.93
-5.589	0.302	2.943	7.608	3.551	-3.787	-6.803	-2.789	108.84

(iii) Monthly minimum temperature (T_{min}) and monthly total rainfall (R_{tot})

Variables								$AICc$
0	R_4	R_5	T_5	T_6	T_7	R_6	R_8	
0.138								266.79
1.549	-6.125							203.50
2.334	-5.657	-2.827						184.77
-0.435	-4.271	-3.509	3.927					175.82
-2.720	-3.467	-3.245	3.963	2.843				167.39
-4.726	-3.370	-3.234	4.748	2.998	2.393			160.83
-2.646	-3.831	-6.277	4.169	2.068	5.501	-7.796		145.04
-1.368	-5.076	-3.238	1.130	0.540	5.077	-9.231	4.109	134.34

(iv) Monthly minimum temperature (T_{min}) and monthly maximum rainfall (R_{max})

Variables										$AICc$
0	R_4	R_5	T_7	R_6	R_8	T_5	T_8	R_3	T_4	
0.138										266.79
1.640	-9.323									205.61
2.378	-6.652	-4.518								169.47
0.159	-10.200	-4.894	4.993							143.309
0.779	-10.306	-6.008	6.708	-5.135						132.57
1.501	-10.529	-6.244	6.450	-4.775	-1.861					130.05
-0.070	-9.798	-5.949	6.592	-4.332	-2.235	1.928				129.90
0.695	-10.564	-6.385	7.791	-5.130	-3.469	2.774	-2.242			127.43
-2.322	-10.738	-6.678	9.695	-1.348	-6.416	6.404	-4.650	3.277		118.64
-3.322	-10.563	-6.598	9.023	-0.552	-6.632	7.145	-5.510	2.882	2.275	117.91

Table S2. The observed and predicted results with 95% confidence intervals in the defined areas using leave-one-out cross-validation. Note that the predicted numbers and upper and lower bounds of confidence intervals were rounded to the nearest integer.

**NTS**	2002	2003	2004	2005	2006	2007	2008	2009	2010	2011	2012	2013	2014	2015	2016	2017	2018
Observed	5	0	0	0	0	0	0	0	0	0	0	0	0	0	0	0	10
Predicted	3	0	0	0	0	0	0	0	0	0	0	0	0	1	0	0	4
CI (lower)	1	0	0	0	0	0	0	0	0	0	0	0	0	0	0	0	1
CI (upper)	7	0	0	0	0	1	0	0	0	0	0	0	1	1	1	0	7

**NTN**	2002	2003	2004	2005	2006	2007	2008	2009	2010	2011	2012	2013	2014	2015	2016	2017	2018
Observed	8	1	0	0	0	0	0	0	0	0	0	0	1	3	0	0	0
Predicted	13	1	0	0	0	1	0	0	1	0	0	0	0	0	0	0	2
CI (lower)	7	0	0	0	0	0	0	0	0	0	0	0	0	0	0	0	0
CI (upper)	21	3	1	0	0	3	0	0	2	0	0	0	0	1	1	0	5

**HKL**	2002	2003	2004	2005	2006	2007	2008	2009	2010	2011	2012	2013	2014	2015	2016	2017	2018
Observed	6	0	0	0	0	0	0	0	4	0	0	0	2	0	4	1	19
Predicted	5	1	0	0	0	2	0	0	0	0	0	0	0	5	0	0	25
CI (lower)	3	0	0	0	0	1	0	0	0	0	0	0	0	2	0	0	16
CI (upper)	9	3	0	0	0	4	0	1	1	1	1	0	1	9	2	0	35

Table S3. The mean squared errors (MSE) and normalised mean squared errors (NMSE) of each model tested using leave-one-year-out (leave-three-out) cross-validation. The MSE and NMSE for the training and the validation sets, and the ratios of the MSE and NMSE of the validation set to that of the training set, respectively, are listed. The best-fitting model was compared with the alternative model (Model: $T_{mean} + R_{tot}$), the fixed effects model (same predictors as the best-fitting model but without random effects) and the full model (all predictors).

	MSE			NMSE		
	Training	*Validation*	*Ratio*	*Training*	*Validation*	*Ratio*
Best-fitting	0.591	7.464	12.629	0.033	0.393	11.559
Alternative	0.650	11.603	17.851	0.037	0.611	16.514
Fixed effects	0.547	7.291	13.329	0.031	0.384	12.387
Full	0.366	>1e04	>1e04	0.021	>1e04	>1e04
